# Injection safety.

**DOI:** 10.3201/eid0707.017714

**Published:** 2001

**Authors:** S Luby

**Affiliations:** Centers for Disease Control and Prevention, Atlanta, Georgia 30333, USA. sluby@cdc.gov


### Injection Safety

**Stephen Luby**
Centers for Disease Control and Prevention, Atlanta, Georgia, USA

Mathematical modeling of available epidemiologic data suggests that each year unsafe injection practices are responsible for 8 to 16 million persons contracting hepatitis B virus (HBV), 2.3 to 4.7 million persons contracting hepatitis C virus (HCV), and 80,000 to 160,000 persons contracting HIV worldwide. In most cases, the transmission of these agents goes unrecognized because the infection is initially subclinical.

An estimated 12 billion injections are administered annually worldwide. Injections are not the most efficient way to transmit HBV, HCV, and HIV, but because so many injections are given, and enough of them are unsafe, they account for a much larger proportion of bloodborne disease transmission than does unsafe transfusion. Global estimates of the percentage of unsafe injections range from 15% in Eastern Europe to 50% throughout Asia.

Injections are popular in many settings because they have important social meaning. Dispensing an injection communicates that the patient's problem is serious and that the provider is concerned and reinforces the special status of the healer in the community. The injection healing ritual usually brings comfort even if it appears irrational from a Western perspective. Because biomedical considerations do not motivate the injection, biomedical concerns with injection safety are often not emphasized.

Safe Injection Global Network (SIGN) was established in October 1999 as a voluntary association of stakeholders who share a common interest in safe and appropriate use of injections. SIGN associates agree to collaborate with other members for developing a common strategic framework and communication strategy. SIGN recommends a three-part, multidisciplinary approach to achieve safe and appropriate use of injections. First, behavior of health care providers and patients must be changed to decrease injection overuse and achieve safety. Second, sufficient quantities of appropriate injection equipment and infection control supplies should be available. Third, a sharps waste management system should be set up to ensure that disposable equipment is destroyed and not reused.

Before SIGN, a number of successful efforts have reduced injection overuse and improved safety. In Indonesia rates of injections decreased from 73% to 14% after group discussions between health care providers and patients. In Tanzania, avoidable injections decreased from 16% to 6% after guidelines to improve injection practices were developed and communicated. In Hafizabad, Pakistan, the proportion of injections conducted with a new sterile syringe increased from 24% to 60% after a health education program was conducted in mosques. Indeed, wherever a plan to promote injection safety has been implemented, it has brought about at least some success.

Vaccine programs are centrally important in promoting safe injections. By adopting safe injection practices, these programs can minimize adverse events resulting from their own intervention. These groups can also share their experience in developing reasonable, safe standards and practices and help catalyze change by being high profile early adopters of injection safety.

Persons interested in learning more about injection safety can visit the Web site www.injectionsafety.org.

Address for correspondence: Stephen Luby, Centers for Disease Control and Prevention, 1600 Clifton Rd., Mailstop A38, Atlanta, GA 30333 USA; fax: 404-639-2205; e-mail: sluby@cdc.gov

